# Letter to the Editor: Advancing deep learning-based segmentation for multiple lung cancer lesions in real-world multicenter CT scans

**DOI:** 10.1186/s41747-025-00649-z

**Published:** 2025-11-05

**Authors:** Xiaowei Huang, Xian Gu

**Affiliations:** 1 Department of Medical Oncology, Seventh People’s Hospital of Shanghai University of Traditional Chinese Medicine, Shanghai, China; 2 Department of Medical Oncology, Longhua Hospital Shanghai University of Traditional Chinese Medicine, Shanghai, China

Dear Editor,

The authors present a valuable contribution toward multi-lesion segmentation in heterogeneous, real-world CT data [1]. However, several issues merit clarification.

First, reporting centers on Dice and detection metrics without explicit, patient-level clinical utility analyses. For multi-lesion disease, downstream decisions depend on lesion counts, longitudinal volume change, and derived radiomics. Aggregation bias can arise when image- or lesion-level gains do not translate to robust patient-level endpoints. Calibration analyses for lesion burden (e.g., calibration plots and Bland–Altman across volume strata), decision-curve analysis for treatment-triggering thresholds, would better ground clinical interpretability [2].

Second, the study spans multi-center data, yet the handling of acquisition heterogeneity (kVp, kernel, slice thickness, contrast timing, inspiratory level) and device-domain shifts is not fully quantified. Without protocol-balanced splits and stratified performance (by vendor, reconstruction kernel, dose, contrast), apparent generalization may reflect inadvertent spectrum bias [3].

Third, sample size accounting at the lesion-level risks pseudo-replication. Multiple lesions per patient and multiple scans per subject introduce clustering that inflates precision if ignored. Confidence intervals and *p*-values should reflect hierarchical structure (patient as cluster, optionally study center as higher level) via mixed-effects or cluster-robust methods. Similarly, when comparing pipeline variants, paired analyses at the patient level with appropriate clustering are preferred over lesion-wise comparisons to avoid unit-of-analysis errors [4].

Fourth, missingness and inclusion pathways are insufficiently detailed. In real-world imaging, exclusions due to corrupted DICOMs, incomplete annotations, atypical fields of view, or motion artifacts can induce selection bias. A flow diagram with counts at each step, plus a comparison of included versus excluded scans on key covariates (age, stage, contrast use, slice thickness), would improve external validity assessment. Multiple imputation or inverse-probability weighting can mitigate bias when covariates are partially missing [5].

Sixth, temporal validation is crucial in oncology care. CT protocols, reconstruction algorithms, and clinical practice evolve over time, and lesion morphology changes with newer therapies. A temporal split (train on earlier years, test on later years) and robustness checks across treatment eras (e.g., targeted therapy, immunotherapy) would address the dataset shift that commonly degrades model performance post-deployment [6].

Seventh, fairness and subgroup robustness deserve explicit analysis. Performance stratified by sex, age, smoking status, histology, and race/ethnicity (where available) can detect disparate error rates. Reporting follows TRIPOD-AI and CLAIM elements only partially; adherence to updated reporting and evaluation guidance (including calibration, decision impact, and fairness) would enhance reproducibility and trustworthiness [7].

In sum, the technical progress is noteworthy. Strengthening statistical design, validation strategy, and reporting along the lines above will better establish real-world safety and utility for multi-lesion lung cancer segmentation.
